# DNA Oxidation and Expression of Repair Enzymes in Organ- Cultured Human Limbal Epithelium

**DOI:** 10.3390/ijms27115073

**Published:** 2026-06-04

**Authors:** Bjørn Otto Nicolaissen, Giang Nguyen, Kahsai Beraki, Amaya Azqueta, Goran Petrovski, Morten C. Moe, Dag Krohn-Hansen, Andrew R. Collins, Bjørn Nicolaissen, Yolanda Lorenzo

**Affiliations:** 1 Department of Ophthalmology, Vestre Viken Hospital Trust, 3004 Drammen, Norway; 2 Center for Eye Research and Innovative Diagnostics, Department of Ophthalmology, Oslo University Hospital, 0450 Oslo, Norway; 3 Faculty of Medicine, University of Oslo, 0372 Oslo, Norway; 4 Department of Pharmaceutical Science, School of Pharmacy and Nutrition, University of Navarra, 31008 Pamplona, Spain; 5 Department of Nutrition, Institute of Basic Medical Sciences, University of Oslo, 0372 Oslo, Norway

**Keywords:** DNA oxidation damage, repair enzymes, human limbal epithelium, 8-oxoG, OGG1, APE1

## Abstract

DNA oxidation damage and its repair are essential for maintaining genomic integrity in the human limbal epithelium, which harbors corneal epithelial stem cells. This study investigated the distribution of the DNA base oxidation 8-oxoguanine (8-oxoG) and the base excision repair (BER) enzymes 8-oxoguanine DNA glycosylase (OGG1) and apurinic/apyrimidinic endonuclease 1 (APE1) in non-cultured and eye-bank organ-cultured human limbal epithelia. Immunohistochemistry was used to assess the localization and staining intensity of 8-oxoG, OGG1, and APE1, evaluated semi-quantitatively and by image analysis. In situ hybridization was performed to detect the distribution of *OGG1* and *APE1* gene expression in organ-cultured tissue. In non-cultured limbal epithelia, nuclear 8-oxoG staining was more frequently observed in superficial epithelial layers, whereas nuclear OGG1 and APE1 staining predominated in basal layers. In organ-cultured epithelia, a higher proportion of superficial nuclei exhibited 8-oxoG staining, while the basal predominance of OGG1 was reduced and that of APE1 was preserved. Transcripts of *OGG1* and *APE1* were detected in basal- as well as in suprabasal layers of organ-cultured epithelia. These findings demonstrate the presence of DNA base oxidation and BER-related enzymes in basal and suprabasal human limbal epithelial cells during storage of corneal tissue under commonly used eye-bank organ-cultured conditions prior to transplantation.

## 1. Introduction

Loss of corneal transparency—and consequently of vision—remains one of the principal causes of blindness worldwide. The global burden is substantial: it is estimated that approximately 5.5 million individuals are either blind or experience significant visual impairment due to corneal opacities [1]. Because the cornea is uniquely positioned at the interface between the external environment and the intraocular structures, maintaining its clarity is essential for proper refraction and retinal image formation. When transparency is compromised, therapeutic options may include transplantation of tissue. Procedures include transplantation of full thickness (penetrating) and of lamellar donor corneal tissue, and of ex vivo engineered tissue.

**Figure 1:**
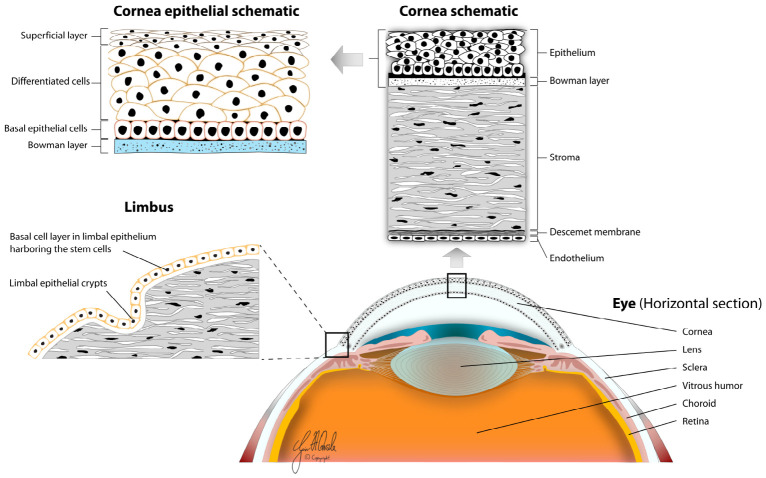
Schematic anatomy of the human cornea and limbal epithelium.

Structurally, the human cornea is composed of five distinct layers arranged from anterior to posterior: the corneal epithelium, Bowman’s layer, the stroma, Descemet’s membrane, and the endothelium. The outermost corneal epithelium is a stratified, non-keratinized squamous epithelium that provides a physical and biochemical barrier against environmental insults while maintaining optical clarity. The epithelium is supported by a thick basement membrane (Bowman’s layer). Beneath this layer lies the collagen-rich stroma, which accounts for approximately 90% of corneal thickness and is essential for mechanical strength. The innermost monolayer of endothelial cells is separated from the stroma by its basement membrane (Descement’s membrane). The endothelium regulates corneal hydration to preserve optical clarity (Figure 1) [2,3,4].

The long-term maintenance and regeneration of the corneal epithelium depend on a specialized population of stem and progenitor cells located in the limbal epithelium, a narrow annular region at the junction between the cornea and the sclera [5]. Limbal epithelial stem cells are responsible for continuous renewal of the corneal epithelium and play a critical role in wound healing and the preservation of corneal transparency [6]. Dysfunction or loss of limbal epithelial cells leads to impaired epithelial regeneration, conjunctivalization, neovascularization, pain, and ultimately loss of vision [7,8]. Consequently, the limbal region represents the key target for regenerative medicine strategies aimed at ex vivo engineering of transplantable epithelial tissue (Figure 1).

Eye banks play a central role in preserving donor corneas during the interval between tissue retrieval and clinical use, and organ-cultured systems allow corneal tissues to retain structural integrity and cellular viability for several weeks [9,10,11,12,13,14,15,16]. In corneal transplantation, donor corneas can be preserved using either hypothermic (cold) storage or organ-cultured methods. Hypothermic storage preserves corneas by keeping them in a specialized solution (e.g., Optisol-GS) at 2–8 °C, which slows cellular metabolism while maintaining endothelial cell viability. The preservation medium contains nutrients, buffering agents, and antibiotics to support tissue health during storage. This method is widely used in North America and several other regions because it is simple and allows short-term preservation (about 7–14 days). Research shows that corneas stored in Optisol-GS at around 4 °C can maintain a high percentage of viable endothelial cells for up to 21 days, demonstrating the effectiveness of this method for transplantation [17,18]. In Europe, donor corneas are routinely preserved using organ culture, which involves storage in a culture medium such as Eagle’s minimum essential medium (MEM) supplemented with fetal bovine serum and antibiotics, typically maintained at 31–37 °C according to standards recommended by the European Eye Bank Association (EEBA). Organ culture allows prolonged storage of corneal tissue, generally up to 28–34 days, while maintaining tissue viability. This preservation method has become the standard approach in most European eye banks because it enables microbiological surveillance of the storage medium, detailed endothelial quality assessment, and safe clinical allocation prior to transplantation. Consequently, organ culture is widely used as it contributes to optimal graft safety and suitability for clinical use [18,19]. The actual storage duration of each donor cornea depends primarily on logistical and clinical factors, including donor availability, microbiological screening procedures, surgical scheduling, and allocation for transplantation. In addition to their use as graft material, donor corneas are increasingly utilized as a source of limbal epithelial cells for ex vivo expansion and the generation of transplantable epithelial sheets, representing an important avenue for regenerative therapies in ocular surface disease [20,21,22,23,24,25].

However, transfer of corneal tissues and cells from their native ocular microenvironment to ex vivo culture systems imposes a series of physiological stresses that can alter redox homeostasis. Ex vivo culture conditions themselves can promote oxidative stress due to factors such as non-physiological oxygen tension, elevated reactive oxygen species (ROS) production, and reduced antioxidant defenses compared with in vivo tissues, resulting in redox imbalance in cultured cells and potential oxidative damage to cellular macromolecules [26,27]. In the context of corneal epithelial cells, primary human corneal epithelial cells cultured under hyperosmolar conditions show significantly increased ROS production and elevated biomarkers of oxidative damage, including lipid peroxidation products (4-hydroxynonenal and malondialdehyde) and oxidized DNA (8-OHdG) compared with standard isosmolar conditions, consistent with oxidative stress responses ex vivo [28]. Several studies have shown that oxidative stress levels in corneal tissues and cells can be influenced by the culture or storage conditions, which may contribute to DNA damage under certain circumstances [29,30,31]. Among the DNA bases, guanosine is particularly vulnerable to oxidative attack [32]. Consequently, the oxidized guanine derivative 8-oxo-7,8-dihydroguanine (8-oxoG) has become a widely used indicator of DNA base oxidation.

The extent of 8-oxoG accumulation reflects not only the magnitude of oxidative insult but also the efficiency of DNA repair pathways responsible for its removal [33]. Base excision repair (BER) is the primary pathway for removing oxidatively damaged bases, particularly 8-oxo-7,8-dihydroguanine (8-oxoG), which is generated by ROS and threatens genomic integrity if unrepaired [34,35]. BER is initiated by lesion-specific DNA glycosylases; in mammalian cells, 8-oxoguanine DNA glycosylase 1 (OGG1) selectively removes 8-oxoG opposite cytosine, preventing mutagenic G→T transversions [35,36,37]. Excision by OGG1 creates an apurinic/apyrimidinic (AP) site, which is processed by apurinic/apyrimidinic endonuclease 1 (APE1). APE1 cleaves the DNA backbone, generating termini for DNA polymerase β and ligases to complete repair while also facilitating OGG1 turnover [38,39].

Deficiency or dysregulation of BER components leads to the accumulation of DNA oxidation damage, mutagenesis, and genomic instability, whereas coordinated *OGG1* and *APE1* activity participate in the preservation of genome integrity [34,40].

Information is scant on DNA base oxidation and on the expression of DNA repair enzymes during organ culture of corneal tissue. In the present study, we used limbal tissues from corneas maintained in eye-bank organ culture and where central corneal tissue had been used for transplant surgery. Our aim, using immunohistochemistry (IHC), was to gather some preliminary information on DNA base oxidation and on the expression and distribution of the BER enzymes OGG1 and APE1.

The limbal epithelium is multilayered, and characteristics differ considerably between cells in the basal- and superficial layers. Therefore, using in situ hybridization (ISH), we considered it of interest to examine whether gene expression of the DNA repair enzyme was localized solely to cells in the basal layer of the organ-cultured epithelium.

## 2. Results

### 2.1. Presence and Distribution of 8-oxoG in Non-Cultured Limbal Tissue and in Organ-Cultured Tissue

**Figure 2:**
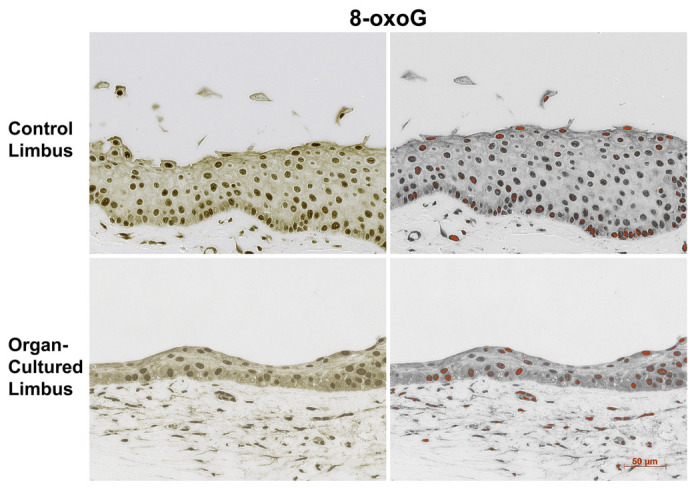
Rows below 8-oxoG show RGB and 8-bit grey photos of DAB staining of sections from non- cultured limbus and from organ-cultured limbal tissue. In the 8-bit grey photos, darkest nuclei show red markers (40×).

To evaluate DNA oxidation damage, immunohistochemistry (IHC) was performed to detect the presence of the DNA base oxidation 8-oxoG in non-cultured limbal tissues as well as in samples maintained in organ culture (Figure 2). Nuclear immunoreactivity for 8-oxoG was evaluated across different epithelial layers, and the intensity of staining was graded semi-quantitatively by three independent researchers to minimize observer bias and increase scoring reliability. 

Using this semi-quantitative approach, we compared the proportion of cells exhibiting high-density staining with those showing only moderate or low reactivity in the superficial, intermediate, and basal epithelial layers. Organ-cultured epithelia displayed an intermediate profile, with roughly 37% of cells in the superficial layers exhibiting high 8-oxoG positivity. In contrast, non-cultured limbal tissue showed a lower proportion of high-density staining, with 20% of superficial nuclei demonstrating strong reactivity.

**Figure 3:**
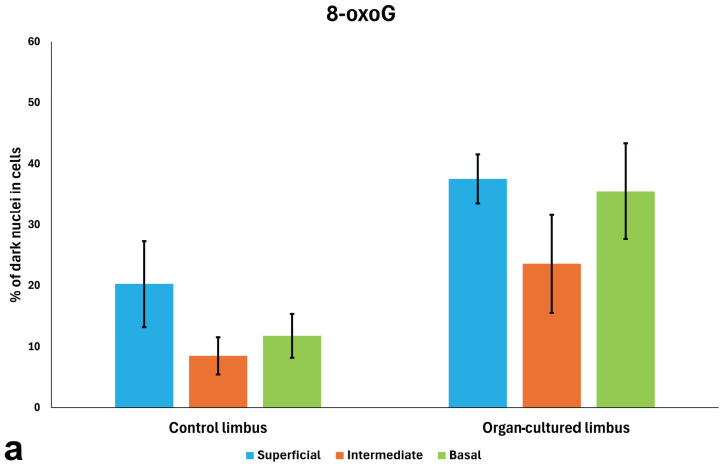

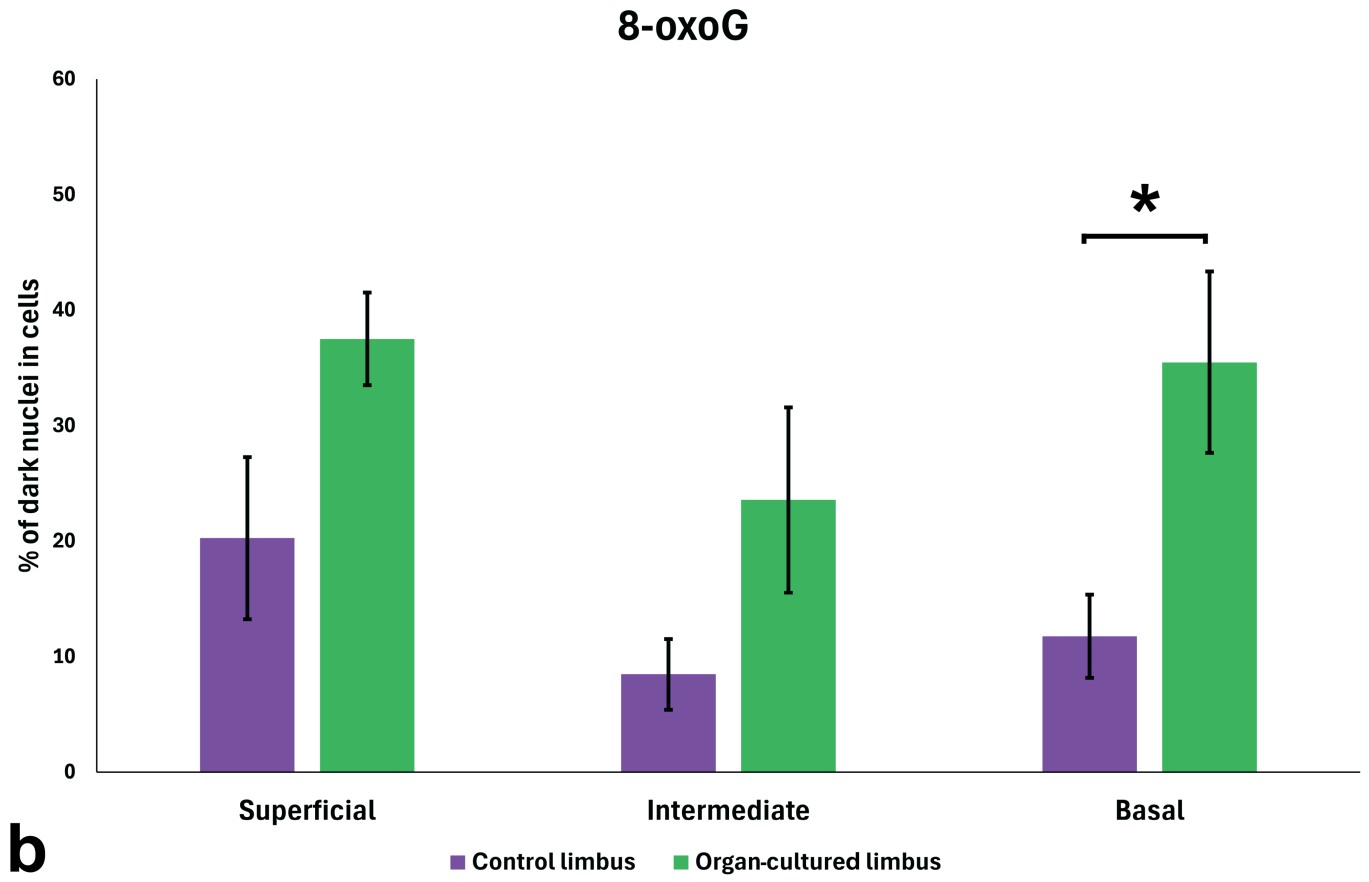
Semi-quantitative evaluation of percentage of dark nuclei in cells stained for 8-oxoG in non-cultured limbus and organ-cultured limbus tissues. Data are presented as the mean percentage of dark nuclei in cells ± SEM (standard error of the mean). (**a**) Comparison of 8-oxoG among the basal, intermediate, and superficial epithelial layers within non-cultured limbus and organ-cultured limbal samples. No statistically significant differences were observed among epithelial layers in either group (Kruskal–Wallis test, *p* > 0.05). (**b**) Comparison of 8-oxoG between non-cultured limbus and organ-cultured limbal tissues within each epithelial layer using the Mann–Whitney U test. A significant increase in 8-oxoG was detected in the basal epithelial layer of organ-cultured limbus compared with non-cultured limbal tissue (*p* = 0.0381), whereas no significant differences were observed in the intermediate or superficial layers (*p* > 0.05). * indicates *p* < 0.05.

In the organ-cultured samples, nuclei with comparable levels of 8-oxoG immunoreactivity were distributed not only in the basal layer but also throughout the suprabasal layers (Figure 3).

Due to the semi-quantitative nature of the scoring system, non-parametric tests were applied. Differences among epithelial layers (superficial, intermediate, and basal) within each group were evaluated using the Kruskal–Wallis test, while comparisons between non-cultured and organ-cultured limbal tissues within each epithelial layer were performed using the Mann–Whitney U test.

No statistically significant differences were observed among the basal, intermediate, and superficial epithelial layers within either the non-cultured limbus or organ-cultured limbal tissue (*p* > 0.05) (Figure 3a).

When comparing non-cultured and organ-cultured tissues within individual epithelial layers, a statistically significant increase in 8-oxoG immunoreactivity was detected in the basal epithelial layer of organ-cultured limbus compared with non-cultured limbal tissue (*p* = 0.0381) (Figure 3b). No statistically significant differences were observed in the intermediate or superficial layers (*p* > 0.05) (Figure 3b).

### 2.2. Expression and Distribution of OGG1 and APE1 in Non-Cultured Limbal Tissue and in Organ-Cultured Epithelium

**Figure 4:**
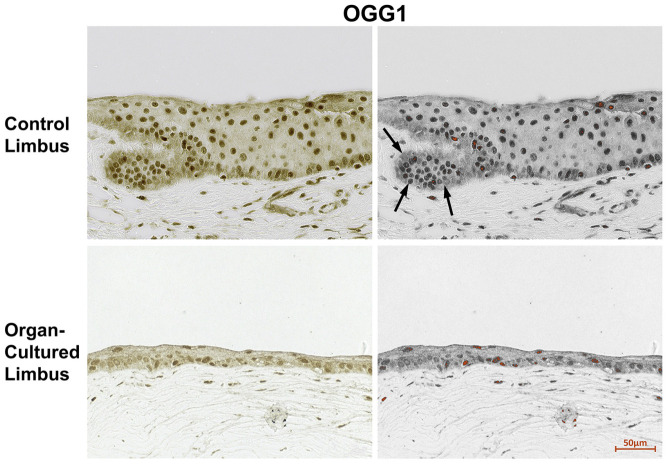
Rows below OGG1 show RGB and 8-bit grey photos of DAB staining for this enzyme in non-cultured limbus and in organ-cultured limbal tissue. In the 8-bit grey photos, darkest nuclei show red markers (40×). Crypt-like formation (arrows).

**Figure 5:**
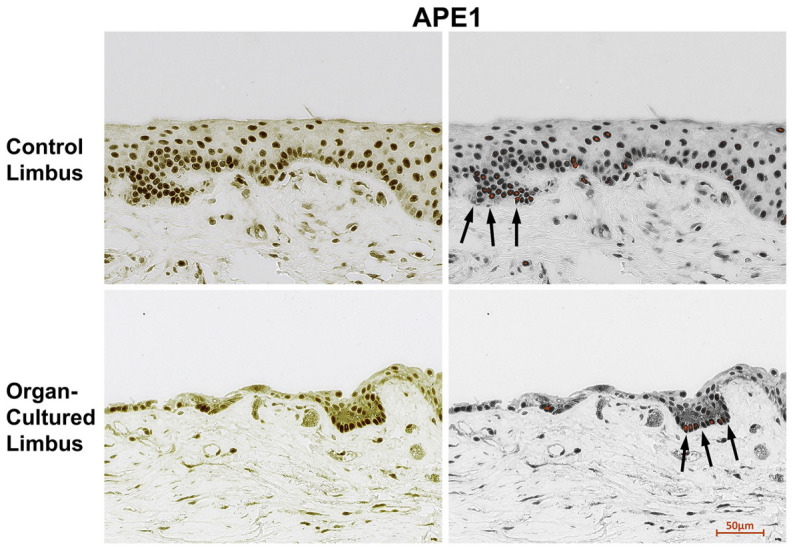
Rows below APE1 show RGB and 8-bit photos of DAB staining for this enzyme in non-cultured limbus and in organ-cultured limbal tissue. In the 8-bit grey photos, darkest nuclei show red markers (40×). High-density staining for APE1 was observed in crypt-like formation (arrows).

IHC was employed to assess the protein-level expression and spatial distribution of the DNA repair enzymes OGG1 (Figure 4) and APE1 (Figure 5) in both non-cultured tissues and in organ-cultured epithelium. In all examined samples, nuclei across the full thickness of the epithelium demonstrated varying degrees of positive immunoreactivity for OGG1. Despite this general pattern, some difference in the distribution of nuclei with high-density reaction was noted between epithelia in non-cultured and cultured samples.

**Figure 6:**
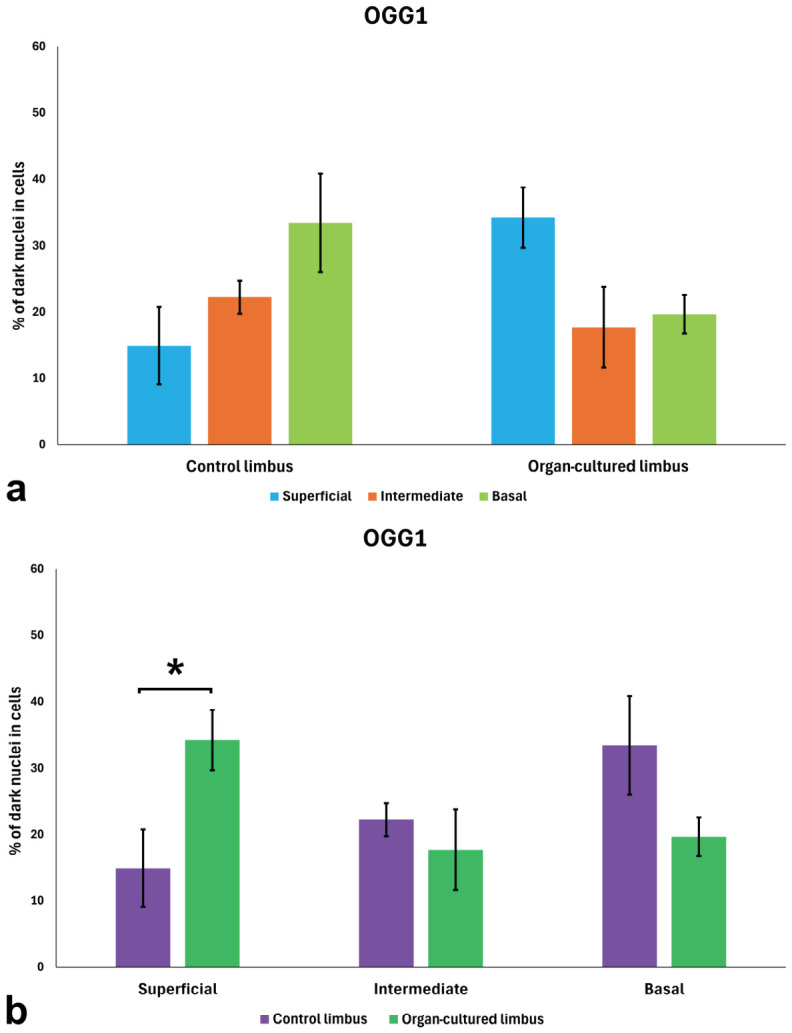
Semi-quantitative evaluation of percentage of dark nuclei in cells stained for OGG1 in non-cultured limbus and organ-cultured limbal tissues. Data are presented as the mean percentage of cells with dark nuclei ± SEM. (**a**) Comparison of OGG1 staining among the basal, intermediate, and superficial epithelial layers within control limbus and organ-cultured limbal samples. No statistically significant differences were observed among epithelial layers in either group (Kruskal–Wallis test, *p* > 0.05). (**b**) Comparison of OGG1 staining between non-cultured limbus and organ cultured limbus tissue within each epithelial layer using the Mann–Whitney U test. A statistically significant increase in OGG1 high-density staining was observed in the superficial epithelial layer of organ-cultured limbus compared with non-cultured limbal tissue (*p* = 0.0286), whereas no significant differences were detected in the basal or intermediate epithelial layers (*p* > 0.05). * indicates *p* < 0.05.

In non-cultured limbal samples, the strongest OGG1 staining was concentrated in the basal epithelial layers, with progressively lower proportions in the intermediate and superficial layers (basal (B) 33%, intermediate (I) 22%, superficial (S) 15%). This gradient was considerably less distinct in organ-cultured samples (B 20%, I 18%, S 34%) (Figure 6).

Kruskal–Wallis testing demonstrated no significant differences among epithelial layers within either experimental group (*p* > 0.05) (Figure 6a).

Mann–Whitney U testing revealed a statistically significant difference in the superficial epithelial layer, where organ cultured limbus samples showed higher OGG1 high-density staining compared with non-cultured limbal tissue (*p* = 0.0286). No statistically significant differences were observed in the basal or intermediate layers (*p* > 0.05) (Figure 6b).

**Figure 7:**
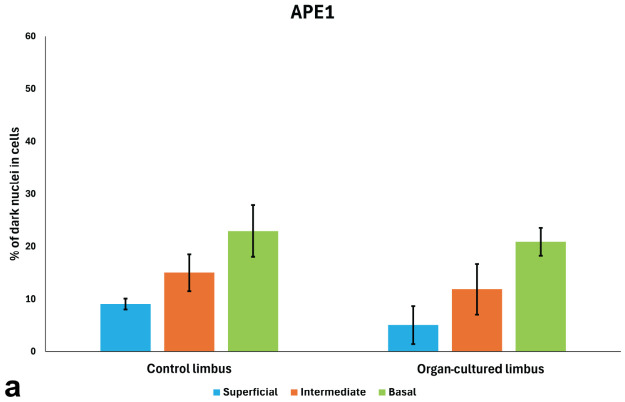

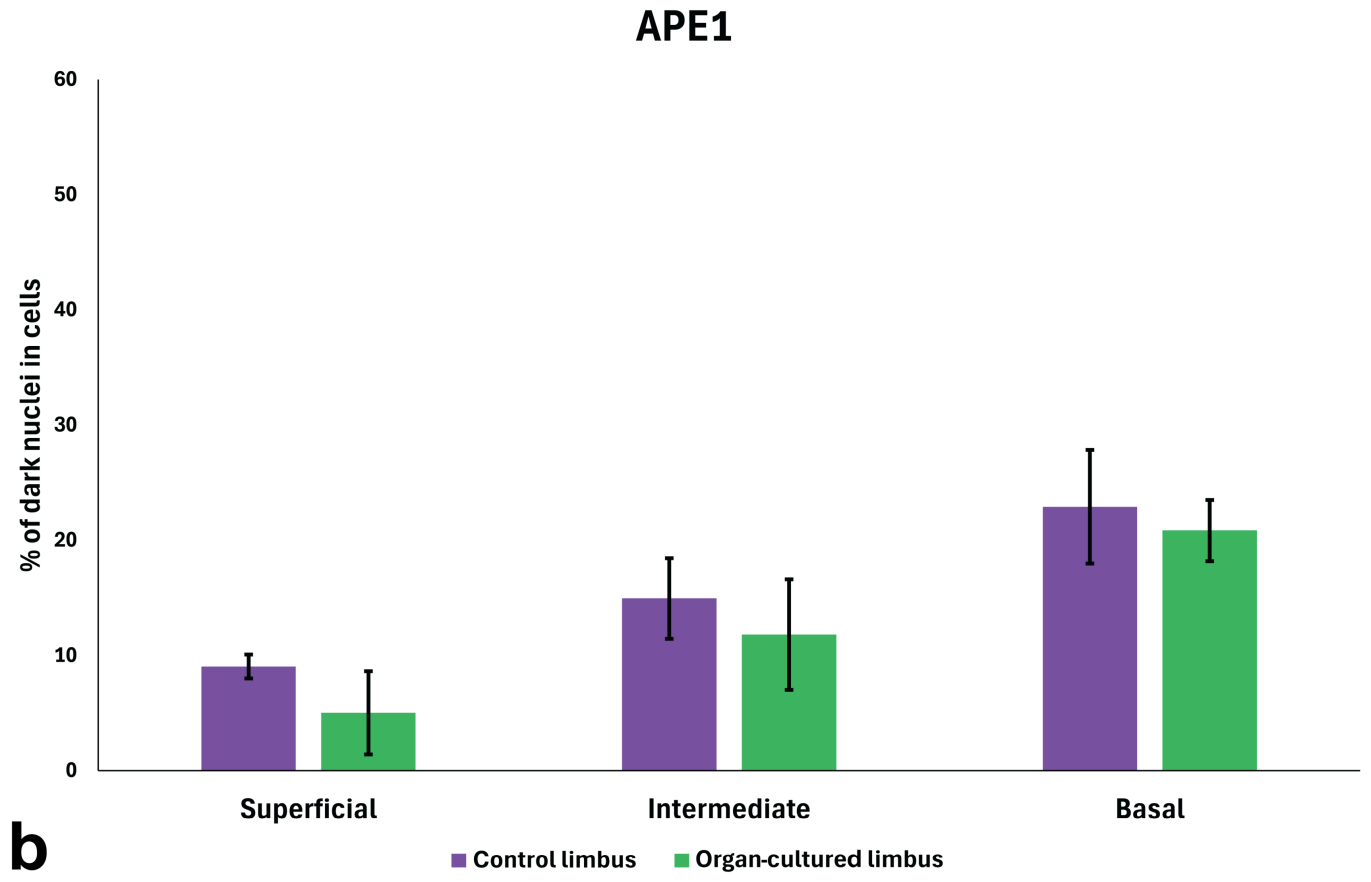
Semi-quantitative evaluation of percentage of dark nuclei in cells stained for APE1 in non-cultured limbus and in organ-cultured limbal tissues. Data are presented as the mean percentage of cells with dark nuclei ± SEM. (**a**) Comparison of APE1 expression among the basal, intermediate, and superficial epithelial layers within non-cultured limbal tissue and organ-cultured limbal samples. No statistically significant differences were observed among epithelial layers in either group (Kruskal–Wallis test, *p* > 0.05). (**b**) Comparison of APE1 expression between non-cultured limbus and organ-cultured limbal tissue within each epithelial layer using the Mann–Whitney U test. No statistically significant differences were observed between groups in the basal, intermediate, or superficial epithelial layers (*p* > 0.05).

High-density staining for APE1 in non-cultured samples also followed a basal-dominant distribution pattern. In the non-cultured limbus, the basal epithelial cells showed the highest proportion of strongly positive nuclei, while intermediate and superficial layers exhibited progressively lower levels (B 23%, I 15%, S 9%). Organ-cultured epithelium demonstrated a similar distribution trend, with high-density staining for APE1 to be most frequently observed in the basal layer (B 21%, I 12%, S 5%) (Figure 7).

Statistical analysis using the Kruskal–Wallis testing revealed no significant differences among epithelial layers within either group (Figure 7a). Similarly, Mann–Whitney U test did not demonstrate significant differences between cultured and non-cultured tissues in any epithelial layer for APE1 expression (Figure 7b).

Notably, both non-cultured limbal tissues and organ-cultured epithelium exhibited distinct clusters of densely stained nuclei for APE1 within crypt-like structures. These formations may represent regions enriched in progenitor or stem-like cells, consistent with functional roles in maintaining genomic integrity in stem cell niches.

**Figure 8:**
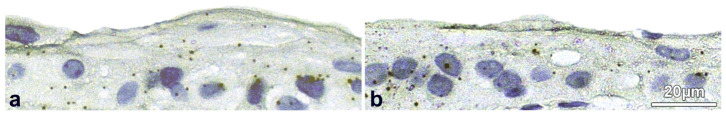
Expression of *OGG1* (**a**) and *APE1* (**b**) were visualized using ISH. Gene transcripts are observed as distinct chromogen precipitates.

Considering the observed distribution of 8-oxoG, OGG1, and APE1 in organ-cultured epithelium, we examined whether *OGG1* and *APE1* expression were restricted to the basal layer. To complement the protein-level analysis, RNA in situ hybridization (ISH) was performed to detect *OGG1* and *APE1*. Expression of both genes was detected throughout all layers of the organ-cultured epithelium (Figure 8).

## 3. Discussion

We detected DNA base oxidation and expression of the base excision repair enzymes OGG1 and APE1 in both non-cultured and organ-cultured human corneal and limbal epithelial samples. Nuclear staining for the DNA base oxidation 8-oxoG was present in all conditions examined, and both repair enzymes were expressed with distinct spatial distributions. In non-cultured tissue, 8-oxoG staining was most prominent in superficial epithelial layers, whereas OGG1 and APE1 showed highest nuclear staining density in basal layers of the limbal epithelium. In organ-cultured epithelium, nuclear 8-oxoG staining was more uniformly distributed across epithelial layers, while the basal predominance of APE1 expression was largely preserved and focal accumulations of APE1-positive nuclei were observed in crypt-like structures.

Previous studies report low levels of DNA oxidation damage in normal corneal epithelium in vivo, with increased damage in pathological conditions such as keratoconus and bullous keratopathy [29,41,42,43,44,45,46]. The dense 8-oxoG staining observed in superficial nuclei of non-cultured samples in the present study may partly reflect post-mortem effects and tissue handling. Although epithelial viability can be maintained for several days after death [11,47], post-mortem changes may alter DNA oxidation damage patterns [48]. Furthermore, the relationship between immunohistochemical staining intensity and absolute levels of DNA oxidative damage is not linear, limiting quantitative interpretation of DAB signal intensity [49,50].

In organ-cultured samples, the relatively uniform distribution of nuclear 8-oxoG across epithelial layers is consistent with previous observations in human corneas maintained in organ culture [31]. Culture conditions are known to influence epithelial phenotype and oxidative stress responses, including medium composition, renewal frequency, and storage system design [16,21,24,51,52]. Closed organ-culture systems may promote oxidative stress due to reduced antioxidant capacity and accumulation of reactive species in the medium [13,30,31], whereas more frequent medium renewal has been associated with lower levels of DNA oxidation damage in limbal epithelial cells [19]. The cytoplasmic 8-oxoG staining observed in addition to nuclear localization may reflect oxidation of mitochondrial DNA, which has been linked to altered cellular function and viability [34,53,54].

Stratified epithelial tissues, including the corneal epithelium, exhibit pronounced polarity and functional heterogeneity between layers. Epithelial renewal is sustained by a population of limbal stem cells located in the basal epithelial layer. Compared with more differentiated epithelial cells in the suprabasal layers, these basal cells possess distinct biological properties, including enhanced mechanisms for coping with cellular stress [55,56,57].

Information on the distribution of OGG1 and APE1 in the human limbal epithelium is limited. In non-cultured limbal epithelium, we observed higher nuclear staining density for both enzymes in basal layers compared with superficial layers, suggesting increased DNA repair capacity in basal cells. In organ-cultured epithelium, this gradient was less pronounced for OGG1 but remained evident for APE1, with clustering of APE1-positive nuclei in crypt-like regions. These findings may reflect differential regulation of base excision repair components during culture and suggest preserved repair activity in regions associated with limbal epithelial maintenance.

Given the observed distribution of 8-oxoG, OGG1, and APE1 in organ-cultured epithelia, we considered it relevant to examine whether expression of *OGG1* and *APE1* were restricted to the basal epithelial layer. Indeed, it was not; expression was present throughout all layers of the epithelium and not confined to basal cells.

Previous ex vivo studies indicate that *OGG1* expression is dynamic and responsive to environmental stressors. For example, exposure of organ-cultured human corneas to atmospheric-pressure cold plasma has been shown to transiently increase *OGG1* expression [58]. APE1 is a multifunctional protein involved in both DNA repair and redox signaling, and altered *APE1* expression has been associated with DNA damage responses and cellular stress [59,60,61].

In conclusion, our findings demonstrate the presence of DNA oxidation damage and expression of base excision repair enzymes in human limbal epithelium on corneal tissue maintained in an eye-bank organ-cultured system commonly used for the preservation of tissue to transplant procedures. Differences in the spatial distribution of 8-oxoG, OGG1, and APE1 between non-cultured and cultured samples may highlight the influence of tissue origin and culture conditions on oxidative stress and DNA repair responses. These observations provide new descriptive data on oxidative DNA damage and repair enzyme localization in the limbal epithelium and support further investigation into how culture systems and storage conditions may affect epithelial genomic integrity.

## 4. Materials and Methods

All experiments were conducted in accordance with the Declaration of Helsinki, and all tissues harvesting was approved by the Local Committees for Medical Research Ethics (REK NR. 1.2007.1099).

### 4.1. Tissue

Human corneoscleral tissue was obtained from rings available after penetrating keratoplasty or automated Descemet’s stripping endothelial keratoplasty and preserved in organ culture prior to use. For age, sex, post-mortem time, and time in organ culture, see Table 1.

The organ-cultured limbal rings (n = 3) were obtained after corneal transplant surgery and were transferred to a 10 cm dishes (Nunclon Surface, Nunc, Denmark) with 15 mL of DMEM/F12 (Invitrogen), and non-cultured limbal tissues (n = 2) were obtained during autopsies as previously described [11]. Peripheral sclera and cornea were trimmed off, and the rings were divided into 12 samples that measured approximately 2 × 2 mm. Samples were washed 3 × 5 min in 15 mL Hanks Balanced Salt Solution without Ca^2+^ and Mg ^2+^ (HBSS) at room temperature.

Samples were fixed immediately in formaldehyde. After that they were dehydrated and embedded in paraffin.

### 4.2. Fixation

Samples were fixed in 4% formalin overnight at 4 °C and then dehydrated in graded alcohol series of 70% (10–15 min), 80% (10–15 min), 96% (2 × 10 min), and 100% ethanol (2 × 10 min), in xylene (3 × 10 min), and embedded in paraffin.

### 4.3. Immunohistochemistry (IHC)

Sections 3.5 µm thick were cut from paraffin-embedded samples using an automated microtome (HM 355S, Thermo Fisher Scientific, Waltham, MA, USA), and sections were attached to histology slides. Deparaffinization was performed in xylene (2 × 10 min). The sections were then rehydrated in series of 100%, 96% and 70% ethanol, and finally in distilled water.

Immunohistochemistry was performed using the following primary antibodies: 8-oxoG (mouse monoclonal 15A3, 1:1500; Santa Cruz, Santa Cruz, CA, USA), OGG1 (rabbit polyclonal, 1:1500; NB100-106, Novus Biologicals, Littleton, CO, USA), and APE1 (mouse 13B8E5C2, 1:2000; Thermo Fisher Scientific, Waltham, MA, USA). Positive immunoreactions were detected using the UltraVision Quanto Detection System HRP DAB kit (Epredia, TL015QHD, Portsmouth, NH, USA), which contains a ready-to-use secondary antibody and DAB chromogen. Slides were mounted with Pertex mounting medium (00840, Histolab, Gothenburg, Sweden). Positive and negative controls were included as recommended by the manufacturer.

### 4.4. Statistical Analysis

Statistical analyses were performed using GraphPad Prism 10 software (GraphPad Software, San Diego, CA, USA). Due to the semi-quantitative scoring system and non-normal data distribution, non-parametric tests were applied. Comparisons between organ-cultured limbus and non-cultured limbal tissues within each epithelial layer were conducted using the Mann–Whitney U test. Differences among epithelial layers within the same experimental group were evaluated using the Kruskal–Wallis test. Data are presented as percentages of cells exhibiting high-density staining. A *p*-value < 0.05 was considered statistically significant.

### 4.5. RNA In Situ Hybridization Assay (ISH)

The assay was performed using RNA-scope 2.5 HD detection kit brown for formalin-fixed, paraffin-embedded (FFPE) tissue (ACD, Hayward, CA, USA) according to the manufacturer’s protocols.

Formalin-fixed, paraffin-embedded tissues were sectioned at 3.5 µm, mounted on SuperFrost Ultra Plus slides (Thermo Fisher Scientific, Waltham, MA, USA) and deparaffinized as recommended by manufacturer Plus, incubated for 30 min at 60 °C to avoid detachment of the sections, incubated with Pretreat One reagent at room temperature for 10 min, boiled with Pretreat Two reagent for 15 min and washed with water. The sections were incubated for 30 min at 60 °C and then pretreated with reagent 3 for 15 min at 40 °C. Samples were hybridized with RNAscope Negative Control Probe 310043, RNAscope Positive Control Probe-Hs-PPIB 313901, RNAscope Probe-Hs-*APEX* 1 480481 and RNAscope Probe-Hs *OGG1* 480391 for 2 h at 40 °C in a humidity chamber in a HybEzTM oven. Following hybridization, slides were washed for 2 min twice in wash buffer, and the signal was amplified using a specific set of amplifiers (Amp) 1–6: Amp 1 for 30 min at 40 °C; Amp 2 for 15 min at 40 °C; Amp 3 for 30 min at 40 °C; Amp 4 for 15 min at 40 °C; Amp 5 for 60 min at room temperature; and Amp 6 for 15 min at room temperature. Washing steps with wash buffer were performed twice between reagents according to the steps indicated above. The signal was detected using DAB for 10 min at room temperature. Slides were counterstained with 50% hematoxylin (Sigma-Aldrich, Saint Louise, MO, USA/GHS116) for 20 s, and bluing was performed with 0.02% ammonia water for 10 s. Slides were washed 3–5 times with water, for 2 min in 70% ethanol, for 2 min in 96% ethanol and for 5 min in xylene. Coverslips were mounted with mounting medium. Images were captured with ZEISS Imager M1 microscope (ZEISS) using 63× magnification.

### 4.6. Images and Evaluation

Images were captured with ZEISS Imager M1 microscope (ZEISS) using 40× magnification. Fraction of nuclei with high-density staining for DAB in basal-, in intermediate-, and in superficial layers of the epithelium was assessed semi-quantitatively by three researchers. Selection of nuclei with high-density staining was assisted by Fiji (ImageJ 1.53f51) whereby the low-level grey value was set at 0 and the upper-level grey value was gradually increased until markers started to appear on nuclei. Graphs and tables showing the mean of relative fractions of nuclei with high-density staining in the epithelia in the different samples are shown in results.

## 5. Conclusions

DNA base oxidation damage and expression of the base excision repair enzymes OGG1 and APE1 are present in both control limbus and organ-cultured human limbal epithelium. In non-cultured limbus tissue, oxidative DNA lesions were most prominent in superficial epithelial layers, whereas nuclear expression of OGG1 and APE1 were highest in basal layers. Organ culture was associated with altered spatial distribution of these markers, indicating that culture conditions may influence the balance between DNA oxidation damage and DNA repair activity.

## Figures and Tables

**Table 1:** Characteristics of the organ culture donor rings obtained for the experiment (age, sex, postmortem time, time of storage in organ culture, time in Cornea MAX and time in Cornea JET).

	1	2	3
**Age**	76	77	41
**Sex**	Male	Female	Male
**Post-mortem time**	20 h	21 h	44 h
**Time of storage in organ culture**	26 days	11 days	15 days
**Cornea MAX**	25 days	10 days	14 days
**Cornea JET**	1 day	1 day	1 day

## Data Availability

Research data are available in publication.

## Abbreviations

The following abbreviations are used in this manuscript:

8-oxoG	8-oxoguanine
BER	Base excision repair
ROS	Reactive oxygen species
OGG1	8-oxoguanine DNA glycosylase
APE1	apurinic/apyrimidinic endonuclease 1
HBSS	Hanks balanced salt solution
IHC	Immunohistochemistry
DAB	Diaminobenzidine
ISH	RNA in situ hybridization assay
FFPE	Formalin-fixed, paraffin-embedded
